# A Competent and Commercially Viable Process for the Synthesis of the Anti-Hypertensive Drug Olmesartan Medoxomil

**DOI:** 10.3797/scipharm.1502-04

**Published:** 2015-03-31

**Authors:** Bommena Hanumantha Rao, Inti Venkata Subramanyeswara Rao, Vysyaraju Ravi Kanth, Korrapati Venkata Vara Prasada Rao, K. Balamurali Krishna, Bethanabatla Syama Sundar

**Affiliations:** 1 Chemical Research and Development, Aurobindo Pharma Pvt Ltd, Survey No. 71& 72, Indrakaran Village, Sangareddy Mandal, Medak District, Telangana-502329, India; 2 Department of Chemistry, Acharya Nagarjuna University, Nagarjuna Nagar, Guntur District, Andhra Pradesh-522510, India

**Keywords:** Olmesartan medoxomil, Process Development, Impurities, Synthesis, Anti-hypertensive Drug

## Abstract

Drug product purity and potency are of most significance in the regulatory market as we notice many recalled batches worldwide, particularly in the US and Japan. Olmesartan Medoxomil is an anti-hypertensive drug. The present invention relates to a process for the preparation of Olmesartan Medoxomil with 99.9% purity in an overall 62% yield. The synthesis includes three isolations and one purification with easy plant operations. This process describes the formation and control of each individual impurity in all stages. This process for Olmesartan Medoxomil and its intermediates is competent for industrial production in very short reaction time intervals with an appreciable yield and high purity.

## Introduction

Hypertension (high blood pressure) is a serious disease with a deleterious impact on health and life expectancy [1]. The renin-angiotensin system (RAS) is one of the most powerful regulators of blood pressure. Up until 1995, angiotensin-converting enzyme (ACE) inhibitors were used, which are drugs capable of blocking the RAS. Later, renin and angiotensin II (A II) receptor antagonists have been developed as specific inhibitors of the RAS [2]. Sartans are a class of drugs that have been developed as specific A II receptor antagonists and there are about seven sartans in clinical practice [3–5]. Olmesartan Medoxomil (**1**) is a nonpeptide angiotensin II receptor antagonist approved by the US FDA in April 2002 for the treatment of hypertension [6]. **1** is a prodrug that rapidly and completely hydrolyses to the active metabolite, olmesartan during gastrointestinal absorption. Head-to-head comparisons with other sartans conducted in the US and Europe have revealed that **1** is superior in lowering blood pressure [2]. The increasing production demands for **1** led to the requirement to redesign the manufacturing process.

Yanagisawa *et al*. described [6] the process for preparation of **1**. In this process, the *N*-alkylation step between the imidazole ethyl ester derivative (**2**) and 4-[2-(trityltetrazol-5-yl)phenyl]benzyl bromide (**3**) have been performed in *N*,*N*-Dimethylacetamide and in the presence of potassium *tert*-butoxide. Ethyl acetate and water were added to the reaction mixture and the product was extracted into ethyl acetate. The purification of the product was achieved by flash column chromatography (ethyl acetate/hexane, 1:2) and an additional crystallization from diisopropyl ether, hexane, ethyl acetate, or in mixtures of them.

Yanagisawa *et al*. disclosed [7] the processes for the preparation of **1**, involving the reaction of (5-methyl-2-oxo-1,3-dioxol-4-yl)methyl 4-(1-hydroxy-1-methylethyl)-2-propylimidazole-5-carboxylate with **3** in *N*,*N*-Dimethylacetamide in the presence of potassium carbonate or reacting **2** and **3** in *N*,*N*-Dimethylformamide in the presence of sodium hydride. In both processes, one commonality was that the alkylated product was subjected to column chromatography in order to obtain an acceptable purity. For the preparation of Trityl Olmesartan Medoxomil (**7**), the product obtained was hydrolyzed by means of an alkali metal hydroxide; the salt was isolated and further esterified with chloro medoxomil. In actuality, the ester hydrolysis suffered from low yield and extraction difficulty, as it solidified during extraction and decomposed at higher temperatures. In the last step, the trityl protection group was removed by treating the Trityl Olmesartan Medoxomil with acetic acid.

Bobba Venkata Siva Kumar *et al*. described [8] the purification processes of **1**, but reported 99.7% purity that contained the Olmesartan acid impurity (**15**).

The general shortcomings of the previous methods reside in the fact that the processes proposed involve, apart from applying column chromatography [6], additional isolation steps [9], which are acknowledged to decrease yield [10] and render any process cumbersome [6, 11, 12]. They also failed to explain the formation and control of side products at each stage [13–18]. Hence, there is a need to perfect the process which could explain the total formation and control of impurities to produce **1** with high purity. During the initial process development of **1**, several routes were examined in detail. The first generation manufacturing process [6] (Scheme 1) was selected for modification on the basis of overall yield, cost, and high throughput.

**Sch. 1:**
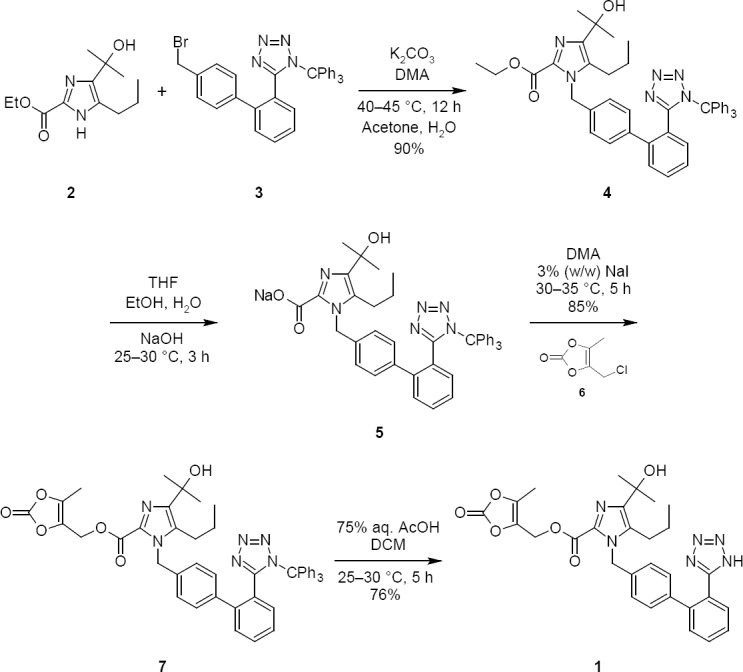
Preparation of Olmesartan Medoxomil (**1**)

## Results and Discussion

The present study was initiated with *N*-alkylation [6] of the imidazole ethyl ester derivative (**2**) which upon reacting with 4-[2-(trityltetrazol-5-yl)phenyl]benzyl bromide (**3**) in the presence of anhydrous K_2_CO_3_ in *N*,*N*-Dimethylacetamide (DMA) afforded Trityl Olmesartan Ethyl Ester (**4**).

The two critical problems encountered in *N*-alkylation were low yield and isolation of compound **4**. The lower yield in the reaction was due to the formation of impurities **5**, **8**, **9**, **10**, **11**, & **12**. The impurities **5**, **8**, & **9** were formed by ester hydrolysis of compounds **2** & **4** (Scheme 2) and the impurities **10**, **11**, & **12** were formed by detritylation of compounds **3** & **4** (Scheme 3), respectively. The impurities **9** & **12** were controlled by specifying or limiting the unreacted compound **3** to no more than 2% at the end of reaction.

**Sch. 2:**
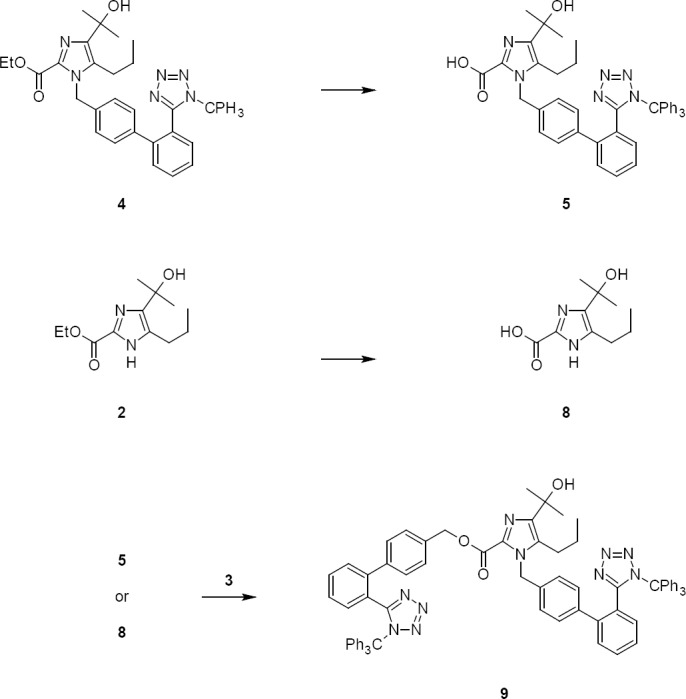
Formation of impurities **5**, **8**, and **9** from **2** and **4**, respectively

**Sch. 3:**
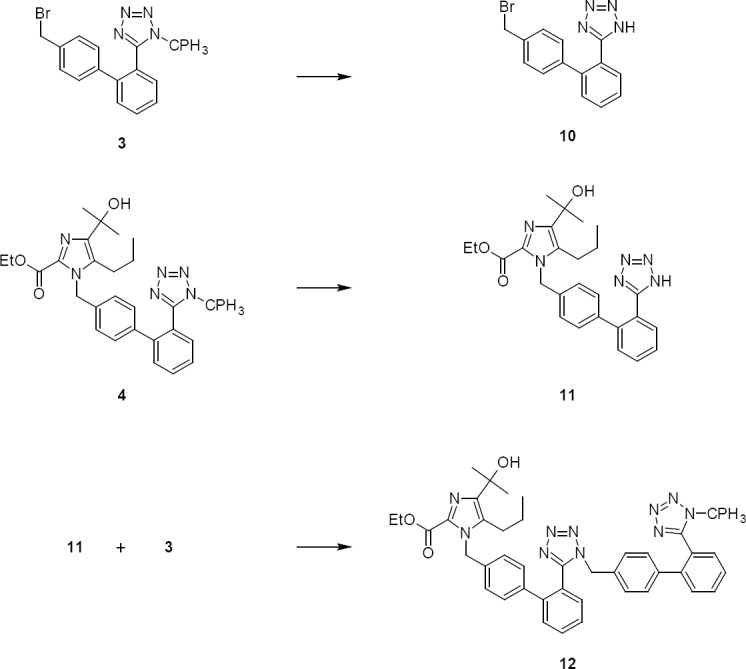
Formation of impurities **10**, **11**, **12** due to detritylation of **3** & **4**, respectively

The specified impurities mentioned should be controlled at this stage or else they may carry forward to the final stage, leading to a lower yield and purity of Olmesartan Medoxomil (**1**).

Herein, we describe the better reaction conditions to control the impurity profile. The *N* alkylation or substitution reaction depends on the mole ratio of potassium carbonate used. To begin, the effect of mole ratio on compound **3** and K_2_CO_3_ on the substitution reaction is studied and the results are summarized in Table 1. The results reveal that the use of 0.98 m.eq of compound **3** and 1.25 m.eq of K_2_CO_3_ in DMA at 40–45°C are suitable conditions as they afforded a 90% yield and 98% purity of compound **4** (Table: 1 entry 6).

**Tab. 1:**
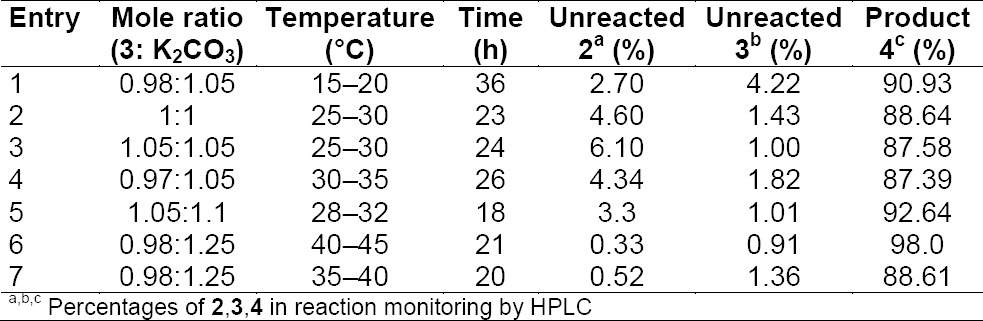
Effects of temperature, mole ratios of **3** and K_2_CO_3_ in the preparation of **4**

The second critical problem is the isolation of compound **4** and to overcome the issue we studied the crystallization of compound **4** in different solvents and different ratios. The results are summarized in Table 2. The different solvent combinations were screened out and the results revealed that the combination of DMA, acetone, and water in the ratio of 3:7:3 was highly suitable for the isolation of compound **4** as it resulted in a 90% yield and 98% purity of compound **4** (Table 2, Entry 7). The advantage of the described process simplifies the plant operations for isolation of the product from the reaction mass which avoids several extractions and distillation.

**Tab. 2:**
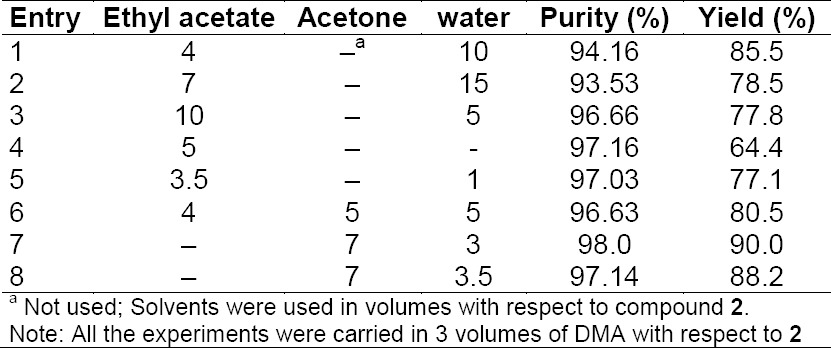
Solvents used in different ratios for isolation of compound **4**

The other advantage of the procedure is cost reduction of the manufacturing process, as the process allows the preparation of **4** from **3** with a purity of 85% which is 30% less expensive than **3** with a purity of 97%. The specified raw material impurities present in compound **3** are methylbiphenyl trityl tetrazole (**13**, ~2 to 5%) and dibromomethylbiphenyl trityl tetrazole (**14**, ~2 to 8%). The crystallized process of compound **4** was successfully executed even after selecting compound **3** with a total raw material impurity of ~9.6%. These impurities were found undetected after the purification process (Table 3).

**Fig. 1:**
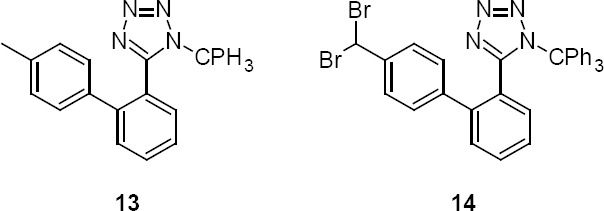
Chemical structures of impurities **13** and **14**

**Table 3:**
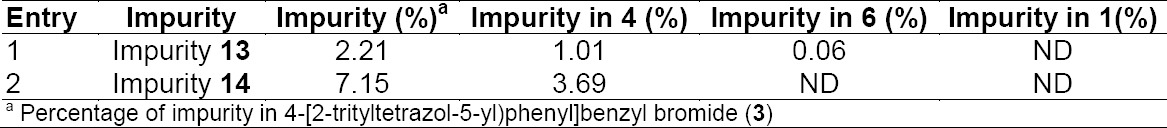
Impurities from **3** and their elimination

This first stage was taken in plant-scale and it was observed that the reaction time intervals were enhanced to 48 h against 24 h as obtained in lab-scale. To overcome the issue, an additional mole ratio of K_2_CO_3_ was incorporated, but this resulted in no difference in reaction time intervals. After a detailed investigation of each and every parameter of the reaction, the reaction time intervals were successfully reduced to 12 h by using K_2_CO_3_ with reduced particle size 200 mesh (90%) from 100 mesh (90%).

The potassium carbonate particle size impact on reaction time is summarized in Table 4.

**Tab. 4:**
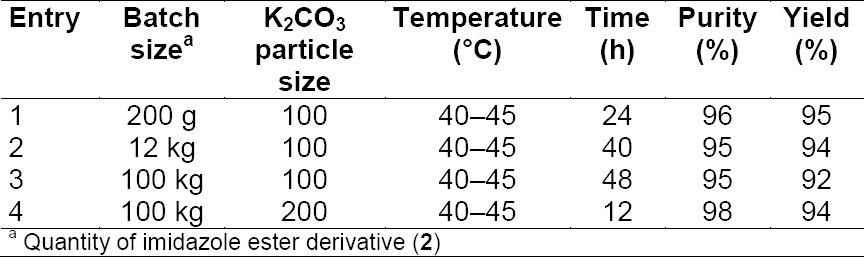
Effect of particle size of K_2_CO_3_ in the preparation of **4**

The stability of **4** was studied at room temperature up to 48 hrs and was found to be stable.

**Tab. 5:**
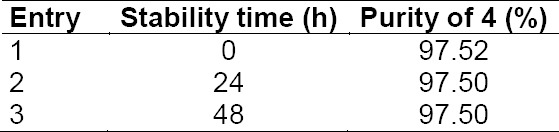
Wet compound stability of **4**

The second stage describes the saponification [6] of compound **4** which afforded compound **5**. The reaction under the specified conditions has [6] not resulted in appreciable purity of compound **5**, leaving 2% of unreacted compound **4** and 5% of impurity **15**. The saponification stage has been evaluated using different mole ratios of NaOH and different solvent ratios and the results are summarized in Table 6. The results revealed that the conditions (Entry 9) are suitable for the preparation of compound **5**. The process afforded the desired purity of compound **5** by minimizing impurities to the NMT 0.5% level. Furthermore, the modified reaction conditions simplified the plant operations by avoiding several extractions and isolations.

**Tab. 6:**
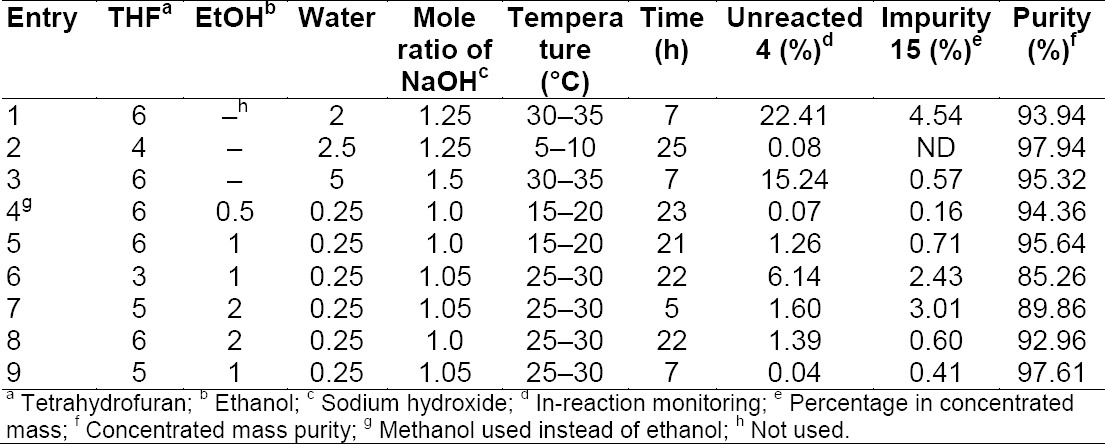
List of different mole ratios of sodium hydroxide in different solvent ratios used for the hydrolysis of **4** to produce **5**

Thereafter, the concentrated mass of **5** upon condensation with **6** produced **7**. As reported in the literature [13], the impurities **15** & **16** were formed in a condensation reaction. The reaction conditions have been modified to overcome the formation of impurities by using a catalytic amount of NaI instead of using base and high temperature conditions. This process afforded an appreciable increase in the yield and purity of compound **7**. The reaction conditions have been tabulated and are summarized in Table 7.

**Tab. 7:**
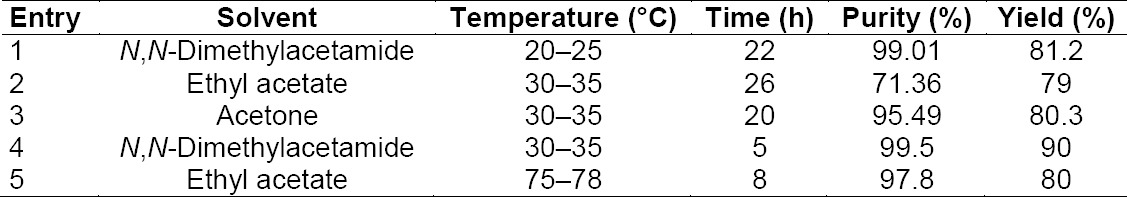
Results of esterification of **5** in various solvents

While all the reaction conditions promoted esterification, their efficiency regarding product yield and purity have proven to be strictly different. The reaction condition with *N*,*N*-Dimethylacetamide as a solvent and catalytic NaI (3% w/w) gave a fair yield and highly pure Trityl Olmesartan Medoxomil (**7**).

The key advantages of using NaI in this esterification rather than base minimized the ester hydrolysis of **7** and detritylation of compounds **5** and **7**. As a consequence, the formation of impurities **15**, **16** as shown in Scheme 4 can be eliminated.

**Sch. 4:**
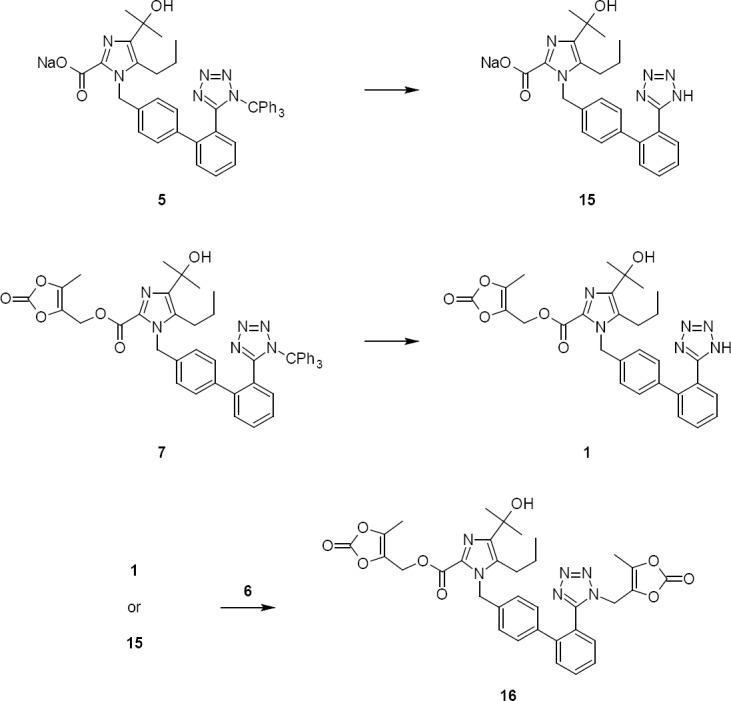
Formation of impurities **15** and **16** during esterification

The stability of wet compound **7** was studied, which confirmed that the product was stable.

**Tab. 8:**
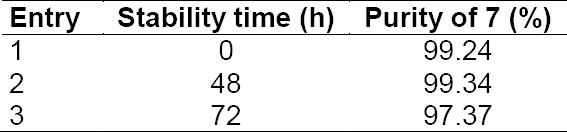
Wet compound **7** stability at ambient temperature

Finally, detritylation [7] of compound **7** using 75% aqueous acetic acid at ambient temperature obtained crude compound **1**. The purification of crude compound **1** was studied in various solvents and the results are summarized in Table 9.

**Tab. 9:**
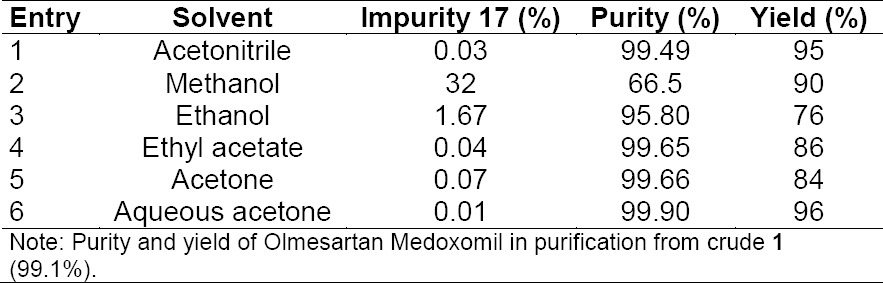
Purification of Olmesartan Medoxomil (**1**) in various solvents and their results

**Fig. 2:**
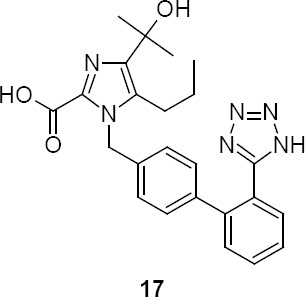
Chemical structure of impurity **17**

Among the solvents used for the recrystallization, it was found that aqueous acetone was the best solvent for purification of **1** which resulted in 99.9% purity in 76% overall yield. As a final point, the total residual solvent levels in **1** were below 1000 ppm and met all the regulatory requirements.

Finally, we disclosed the reinvestigation efforts [19] of the process for making Olmesartan Medoxomil (**1**, ≥99.9%) in industrial scale. The results include the reinvestigation of the described process as well as its novel modifications such as N-alkylation with anhydrous K_2_CO_3_ with reduced particle size, and the easy isolation procedure to get the pure Trityl Olmesartan Ethyl Ester in 90% yield. Saponification followed by esterification in the presence of 3% (w/w) NaI resulted in Trityl Olmesartan Medoxomil in 90% yield with ≥99.5% purity by HPLC. Total synthesis included less reaction time and 62% overall yield to get 99.9% pure Olmesartan Medoxomil using easy plant operations. In addition, the formation and control of each individual impurity at every stage was described.

## Experimental

^1^H-NMR, ^13^C-NMR, and spectral data were performed in dimethyl sulfoxide (DMSO-*d_6_*) with 300 MHz spectrometers. The chemical shift values were reported on the δ scale in parts per million (ppm), downfield from tetramethyl silane (TMS, δ = 0.0) as an internal standard. Spin multiplicities are given as s (singlet), d (doublet), dd (doublet of doublet), t (triplet), and m (multiplet) as well as brs (broad). Coupling constants (*J*) are given in hertz. IR spectra were recorded in the solid state as KBr dispersions using a Perkin-Elmer Spectrum One Fourier Transform (FT)-IR spectrophotometer. The mass spectrum was recorded using a Perkin-Elmer PE SCIEX-API 2000, equipped with an ESI source used online with a HPLC system after the ultraviolet (UV) detector. HPLC chromatographic purity was determined by using the area normalization method. The thermal analysis was carried out on a DSC Q 1000 TA. The thermogram was recorded from 40 to 320°C. The solvents and reagents were used without purification.

### Preparation of Trityl Olmesartan Ethyl Ester (4) Ethyl 4-(2-hydroxypropan-2-yl)-2-propyl-1-({2’-[1-(triphenylmethyl)-1H-tetrazol-5-yl][1,1’-biphenyl]-4-yl}methyl)-1H-imidazole-5-carboxylate

To a solution of **2** (100 kg, 416.6 mol) in *N*,*N*-Dimethylacetamide (300 L) was added powdered anhydrous potassium carbonate (72 kg, 521.7 mol) followed by **3** (227.5 kg, 408.4 mol) at 25–30°C. Thereafter, the reaction mass temperature was raised to 40–45°C and stirred for 12 h. After completion of the reaction, acetone (700 L) was added to the reaction mass at 35-40°C, which resulted in a slurry mass. To this slurry mass, DM water (300 L) was added in 15 min at 20–25°C and cooled to 0–5°C to afford **4** (268 kg, 90%) as a white crystalline solid with 98% purity by HPLC. Mp 164–167°C. IR (KBr): 3403, 3088, 3055, 3026, 1778, 1701, 1666, 1524, 1492, 1469, 1446, 1409, 1396, 1376, 1335, 1335, 1176, 1142, 1055, 1032, 928, 778, 746 cm^-1^. ^1^H-NMR (300 MHz, DMSO-*d_6_*): δ = 7.75–6.83 (m, 23H), 5.41 (d, 3H), 4.06 (q, 2H), 2.45 (d, 2H), 1.52 (m, 8H), 0.99 (t, 3H), 0.77 (t, 3H). MS: *m/z* = 717.6 [M+H] ^+^.

### Preparation of Trityl Olmesartan Medoxomil (7) (5-Methyl-2-oxo-2H-1,3-dioxol-4-yl)methyl 4-(2-hydroxypropan-2-yl)-2-propyl-1-({2’-[1-(triphenylmethyl)-1H-tetrazol-5-yl][1,1’-biphenyl]-4-yl}methyl)-1H-imidazole-5-carboxylate

To a pre-cooled solution of **4** (200 kg, 279.3 mol) in a mixture of tetrahydrofuran (1200 L), ethanol (200 L) was added to a pre-cooled aqueous sodium hydroxide (11.73 kg, 293.2 mol) solution in DM water (50 L) at 10–15°C and stirred for 5 h. Thereafter, we concentrated the reaction mass to below 20°C under reduced pressure to afford Trityl Olmesartan sodium salt (**5**) as a thick oily mass. To the solution of **5** in DMA (600 L) was added 4-(chloromethyl)-5-methyl-1,3-dioxol-2-one (**6**) (50.69 kg, 90% purity by GC, 307.2 mol) and sodium iodide (6 kg, 3% w/w with respect to **4** at 25-30°C. The contents were heated and stirred for 5 h at 30–35°C. To the resulting clear solution was added ethyl acetate (2000 L), DM water (2000 L), followed by sodium metabisulphite (2 kg) at 30–35°C and stirred for 15 min. The layers were separated and the organic layer was washed with 20% w/w aqueous sodium chloride solution (2×1000 L) at 30-35°C. The organic layer was concentrated at 30–40°C under reduced pressure till the distillate volume was 1150 L, and then diisopropylether (1000 L) was added at 30–40°C. The slurry was cooled to 0–5°C and stirred for 30 min. The product was filtered and dried under reduced pressure to obtain **7** as a white powder (203 kg, 90%) with 99.47% purity by HPLC. Mp 104–106°C. IR (KBr): 3398, 3059, 3027, 2873, 1819, 1805, 1737, 1707, 1527, 1492, 1465, 1393, 1357, 1256, 1170, 1094, 1004, 726, 678 cm^-1^. ^1^H-NMR (300 MHz, DMSO-*d_6_*): δ = 7.78–7.29 (m, 13H), 7.02 (d, 2H), 6.87 (d, 6H), 6.77 (d, 2H), 5.35 (s, 2H), 5.25 (s, 1H), 5.00 (s, 2H), 2.43 (t, 2H), 2.02 (s, 3H), 1.54 (m, 2H), 1.49 (m, 6H), 0.75 (t, 3H). MS: *m/z* = 801.4 [M+H]^+^.

### Preparation of Olmesartan Medoxomil (1) (5-Methyl-2-oxo-2H-1,3-dioxol-4-yl)methyl 4-(2-hydroxypropan-2-yl)-2-propyl-1-{[2’-(1H-tetrazol-5-yl)[1,1’-biphenyl]-4-yl]methyl}-1H-imidazole-5-carboxylate

The suspension of **7** (175 kg) in 75% v/v aqueous acetic acid (875 L) was stirred at 25–30°C for 10 h. The byproduct, trityl alcohol, was filtered through hyflo and washed with 75% v/v aqueous acetic acid (200 L). Methylene chloride (1225 L) was added to the filtrate followed by DM water (875 L), which was added at 20–30°C and stirred for 15 min. The layers were separated and the aqueous layer was extracted with methylene chloride (525 L) at 20–30°C. The combined organic extract was washed with DM water (2×1750 L) at 20-30°C. Water was added at 20–30°C to the organic layer and its pH was adjusted to 7.30–7.50 with 5% w/w aqueous sodium bicarbonate (4.3 kg in 81 L of Dm water) at 20–30°C. The organic layer was separated and concentrated at 30-40 °C under reduced pressure. Acetone (700 L) was added to the concentrated mass and distillation was continued at 50–55°C at ambient pressure, till the distillate volume was 600 L. The resulting slurry was cooled to 0–5°C and stirring was continued for 1 h. The product was filtered and dried to afford **1** (109 kg, 89%) with 99.7% purity by HPLC. Mp 182–184°C. IR (KBr): 3290, 3039, 3003, 2930, 1922, 1832, 1707, 1551, 1474, 1389, 1301, 1258, 1225, 1168, 1135, 1088, 1002, 953, 816, 782, 760 cm^-1^. ^1^H-NMR (300 MHz, DMSO-*d_6_*): δ = 7.67 (m, 2H), 7.56 (m, 2H), 7.04 (d, 2H), 6.86 (d, 2H), 5.42 (s, 2H), 5.32 (brs, 1H) 5.24 (s, 2H), 2.62 (t, 2H), 2.08 (s, 3H), 1.59 (q, 2H), 1.47 (s, 6H), 0.88 (t, 3H). MS: *m/z* = 559.3 [M + H]^+^.

#### Purification of Crude Olmesartan Medoxomil (1)

Olmesartan Medoxomil crude (94 kg), carbon (9.4 kg, 10% w/w) was added to acetone (1600 L) and we heated the contents to reflux temperature, and stirring was continued for 30 min. The contents were cooled to 45–50°C and the carbon was filtered through hyflo, washed with hot acetone (188 L, 50°C). Ethyl acetate (94 L) was added to the filtrate and concentrated to below 55–60°C at ambient pressure to collect 1560 L of the distillate. Thereafter, the resulting slurry was cooled to 0–5°C and stirred for 30 min. The product was filtered and dried to afford pure Olmesartan Medoxomil (84.6 kg, 90%) as white crystalline powder having 99.9% of HPLC purity.

The obtained Olmesartan Medoxomil (80 kg) was suspended in DM water (1600 L) at 25–30°C and stirring was continued for 1 h at this temperature. The product was filtered and dried under reduced pressure to afford pure **1** (76.8 kg, 96%) having residual acetone less than 1000 ppm.
